# Spatially Profiling Trace Cytokine Signatures From Microscopically Derived Skin Samples to Probe Skin Disease Inflammation

**DOI:** 10.1002/smtd.202501964

**Published:** 2026-05-12

**Authors:** Aditya Krishnakumar, Zhen Zhang, Courtney Vedelago, Timothy J. Liu, Yung‐Ching Kao, H. Peter Soyer, Mitchell S. Stark, Snehlata Kumari, Matt Trau, Alain Wuethrich

**Affiliations:** ^1^ Centre For Personalised Nanomedicine Australian Institute For Bioengineering and Nanotechnology (AIBN) The University of Queensland Brisbane Queensland Australia; ^2^ Frazer Institute The University of Queensland Dermatology Research Centre Brisbane Queensland Australia; ^3^ Department of Dermatology Princess Alexandra Hospital Brisbane Queensland Australia; ^4^ School of Chemistry and Molecular Biosciences The University of Queensland Brisbane Queensland Australia

**Keywords:** inflammatory skin diseases, nanomedicine, psoriasis, single molecule detection, surface enhanced Raman spectroscopy

## Abstract

Inflammatory skin diseases, such as atopic dermatitis, hidradenitis suppurativa and psoriasis, involve chronic aberrations to innate and adaptive immunity, driven by pro‐inflammatory cytokines. Various minimally invasive methods exist that allow for proteomic analysis of affected skin lesions in microscale concentrations. However, analyzing cytokines in small skin samples using traditional immunoassays is challenging due to many cytokines being detectable only at pico‐scale concentrations. Here, a digital surface‐enhanced Raman spectroscopy (SERS) immunoassay has been developed for multiplex detection of IL‐17A, IL‐22, IL‐23 and TNF in punch biopsy skin using psoriasis as a model. It is performed using single‐molecule counting via analyte compartmentalization on a nanopillar array and single‐particle active SERS nanotags. This SERS nanopillar assay demonstrated a high degree of specificity and sensitivity, detecting cytokines down to 104 aM. Promisingly, this assay successfully detected cytokines in microscopically derived skin punch biopsy samples containing 13.84 µg to as little as 0.34 µg of total protein, revealing distinct cytokine profiles. The assay was also able to show the differences in cytokine levels between perilesional and lesional psoriasis skin samples. The high detection sensitivity of the digital SERS nanopillar assay, combined with cytokine profiling in biopsy samples, shows promise for sensitive immunological diagnosis and therapeutic monitoring of inflammatory skin diseases.

## Graphical abstract

Inflammatory skin diseases are driven by immune dysregulation and cytokine imbalance. A challenge persists in profiling cytokines in microscopically derived biopsy skin samples due to low protein content. This study introduces a digital SERS immunoassay to simultaneously detect IL‐17A, IL‐22, IL‐23, and TNF at trace concentrations as low as 104 aM. The assay achieved single‐molecule sensitivity and is capable of distinguishing cytokine levels at different psoriasis skin lesion sites.

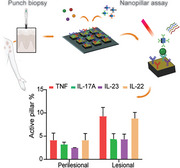

## 1 Introduction

Inflammatory skin diseases significantly impact the quality of life of affected individuals by causing physical discomfort, emotional distress, and social stigmatization [1]. According to the World Health Organization's Global Psoriasis Report, the global incidence of psoriasis has risen significantly and is estimated to affect 2%–3% of the population worldwide [2]. In psoriasis, diagnostic latency remains a well‐documented concern particularly for psoriatic arthritis where delays commonly range from 1.6 to over 2 years before definitive diagnosis and intervention [3]. Psoriasis is a highly complex disease influenced by multiple factors, including genetic predisposition, infections, immune dysfunction, and mechanical trauma [4]. This highlights a critical need for personalized treatment strategies to improve patient outcomes.

Psoriasis is driven by dysregulation of epithelial and immune cell functions [5]. It involves abnormal keratinocyte proliferation, inflammatory cell infiltration, and dysregulated cytokine signaling [6]. Several biologics are being used for the treatment of psoriasis with the most successful targeting interleukin 23 (IL‐23) and T helper 17 (Th17) [7, 8]. Interleukin 17 (IL‐17) as a key cytokine in psoriasis pathogenesis, establishes a self‐perpetuating inflammatory loop in keratinocytes, promoting excessive proliferation and further immune cell recruitment [9, 10]. Moreover, myeloid cells secreting IL‐23 stimulate Th17 and T helper 22 (Th22) differentiation and trigger the release of Tumor necrosis Factor (TNF), IL‐17, and interleukin 22 (IL‐22) [11]. IL‐17A, IL‐22, IL‐23, and TNF serve as valuable biomarkers for disease severity and progression [12], however the stratification of patients who would react to one or the other cytokine inhibition is currently unknown. Hence, measuring these cytokines in affected tissues through skin biopsies throughout the treatment can improve diagnosis and inform personalized targeted immunomodulatory therapies [13, 14, 15].

Inflammatory skin lesions are commonly diagnosed through histological examination of 2–4 mm punch biopsy of the affected lesional skin, with or without perilesional skin by pathologists [16, 17]. However, this method requires local anesthesia and often sutures, making it invasive and unsuitable for frequent sampling [18]. To address this limitation, several minimally invasive skin sampling techniques have been developed. Tape stripping is one such method that involves sequential removal of the stratum corneum using adhesive films [19, 20, 21, 22]. Meanwhile, microbiopsy sampling, a more recent innovation, extracts approximately 0.15 mm diameter of tissue from skin without the need for any anesthesia [23]. Detecting cytokines in cutaneous samples is a promising avenue to improving accurate diagnosis and enabling timely, targeted interventions. Specifically, through identifying pathognomonic inflammatory signatures, which may be targeted by biologics and other advanced therapeutics [24, 25]. However, the detection of cytokines is challenging due to limited tissue volume and the complexity of the sample matrix, necessitating highly sensitive and specific assays [23]. Additionally, conventional techniques such as immunoassays, immunohistochemistry, and mass spectrometry, though widely used, often fall short in quantifying cytokines at low concentrations with sufficient accuracy and reproducibility [26, 27].

To overcome these limitations, emerging technologies incorporating nanomaterials and microfluidic platforms have been developed to enhance sensitivity and enable multiplexed detection in small‐volume samples [28, 29, 30]. For example, nanostructured plasmonic substrates and advanced signal amplification strategies have been reported to enhance detection sensitivity and multiplexing capabilities in various biosensing applications [31, 32, 33, 34, 35]. Nanomaterial‐based biosensors utilizing gold nanoparticles, quantum dots, and carbon nanostructures offer superior signal amplification and are well‐suited for trace‐level cytokine detection [37, 38]. Among these, surface‐enhanced Raman scattering (SERS) has shown exceptional promise. SERS is a highly sensitive spectroscopic technique capable of detecting molecules down to the single‐molecule level [39]. It leverages plasmonic nanostructures typically composed of metallic substrates conjugated to Raman reporter molecules, which enhance Raman scattering signals when excited by a specific wavelength [40]. This signal amplification occurs through localized surface plasmon resonance, dramatically increasing the Raman signal intensity. By combining the molecular specificity of Raman spectroscopy with the signal enhancement provided by these nanostructured metallic substrates, SERS enables ultra‐sensitive and multiplexed detection of biomarkers. This makes it an especially promising method for analyzing low level cytokines in skin obtained from microsampling of patients with inflammatory skin diseases, such as psoriasis.

In this study, we developed a digital SERS nanopillar assay for ultrasensitive and multiplexed detection of IL‐17A, IL‐23, IL‐22, and TNF in skin biopsy samples. While the core sensing principle and nanopillar architecture have been reported previously [40], this study presents the first proof‐of‐concept application of the digital SERS nanopillar platform for cytokine detection of extremely low‐input from human skin biopsy samples. In addition, the platform enables multiplex cytokine profiling in paired lesional and perilesional samples, supporting its potential for spatially resolved immune analysis. We demonstrate the capability of our digital SERS nanopillar assay to consistently detect and quantify trace cytokines, hence enabling the profiling of cytokines in 2 mm skin biopsy in both lesional and perilesional skin samples. Furthermore, the novelty of this study is the ability to detect distinct cytokine profiles in low abundance, which might establish a proof‐of‐concept for integrating microsampling techniques and SERS nanopillar assay into routine clinical practice. This advancement opens avenues for highly sensitive cytokine monitoring, supporting personalized treatment strategies.

## 2 Materials and Methods

### 2.1 SERS Nanopillar Chip Fabrication

The nanopillar chip measured 1 mm × 1 mm and contained approximately 250 000 pillars. Each pillar was 1 µm in width, 1 µm in length, and 1 µm in height, with a spacing of 1 µm between adjacent pillars. The nanopillar chip was fabricated on a 4‐inch silicon wafer through a stepwise process involving electron beam lithography, electron beam metal evaporation, and reactive ion etching, as reported previously [41].

### 2.2 Synthesis of SERS Nanotags

The SERS nanotags were synthesized according to our previous report [34, 35]. Briefly, using a mixture of gold chloroauric acid (HAuCl_4_) and silver nitrate (AgNO_3_) in Milli‐Q water, followed by surface functionalization via DSP (dithiobis (succinimidyl propionate)) linkage to ensure a strong antibody conjugation to Raman reporters. The Raman reporters used for cytokine identification were 2,7‐mercapto‐4‐methylcoumarin (MMC) for IL‐22, 5,5′‐dithiobis (2‐nitrobenzoic acid) (DTNB) for IL‐17A, 2,3,5,6‐tetrafluoro‐4‐mercaptobenzoic acid (TFMBA) for IL‐23, and 4‐mercaptobenzoic acid (MBA) for TNF. Raman reporters were selected based on their distinct and well‑resolved characteristic peaks. Specifically, MBA, MMC, TFMBA, and DTNB were chosen because they exhibit characteristic Raman peaks at 1080, 1175, 1380, and 1330 cm^−1^, respectively, with minimal spectral overlap [42, 43]. Individual reporter‐labelled nanotags were characterized prior to multiplex measurements to confirm minimal spectral overlap, and reporter‐specific peak positions were used for signal extraction. Initially, 1 mL of nano box suspension was centrifuged at 800 g for 15 min and resuspended in 200 µL of Milli‐Q water. The nano boxes were then incubated at 25°C and 350 rpm for 6–8 h with a mixture of Raman reporters 15 µL/200 µl MMC (1 mm), 15 µL/200 µl MBA (1 mm), 15 µL/200 µl DTNB (1 mm), or 15 µL/200 µl TFMBA (1 mm) along with 2.5 µL/200 µl 1 mm of DSP, which served as a linker molecule. After incubation, the solution was centrifuged again (800 g, 15 min) to remove unbound Raman reporters and DSP, followed by antibody functionalization. Specifically, 1 µL (0.5 µg) of antibody was added to the MMC‐, DTNB‐, and TFMBA‐conjugated nano boxes, while 0.4 µL (0.2 µg) was added to the MBA‐conjugated ones. The mixtures were incubated for another 30 min at 25°C, 350 rpm. Excess antibodies were removed via centrifugation, and the nanobox surfaces were then blocked with 200 µL of 0.1% BSA to prevent nonspecific binding. The SERS nanotags with MBA, MMC and DTNB Raman reporters were diluted 5‐fold in 1X phosphate‐buffered saline (PBS), while SERS nanotags with TFMBA were diluted 3‐fold in 1X PBS prior to use.

### 2.3 Functionalization of Nanopillar Assay

Nanopillar chips were prepared as described in the previous report [35]. After fabrication, the chips were washed three times with isopropanol and blown dry with a stream of nitrogen. A 4 mg mL^−1^ solution of DSP in Dimethyl sulfoxide (DMSO) was prepared, and 10 µL was applied to each chip, followed by 30 min of incubation at room temperature. Post‐incubation, chips were sequentially washed with isopropanol, deionized water, and PBS. Subsequently, 10 µL PBS containing 5 µg mL^−1^ of antibodies against IL‐17A (R&D Systems, Cat no: AF317‐NA), IL‐22 (R&D Systems, Cat no: AF782), IL‐23p19 (R&D Systems, Cat no: AF1716), and TNF (R&D Systems, Cat no: MAB610) were incubated on the chip for 2 h at room temperature. To block non‐specific binding, 10 µL of 2.5% BSA in PBS was added without washing off the antibody, followed by a 1 h incubation at room temperature. Chips were then washed with a 0.5% Tween 20 wash buffer, followed by two washes with 1X PBS to remove excess bioreagents.

### 2.4 Patient Sample Collection and Preparation

Skin samples were collected via 2 mm punch biopsy from three participants with chronic plaque psoriasis presenting to the Department of Dermatology, Princess Alexandra Hospital as described previously [44]. All participants received paired perilesional and lesional biopsies, and the anatomical sites differed between the participants.

In this study, the biological replicates (*n* = 3) were performed with triplicate technical replicates on the chip. Biopsies were homogenized in PBS containing a protease inhibitor cocktail to preserve cytokine integrity [44]. Lysates were clarified by centrifugation and stored at −80°C until analysis. Protein concentration in skin homogenate was measured by Pierce BCA Protein Assay (Thermo Fisher, Cat no. A65453) according to the manufacturer's protocol.

This research involved the recruitment of patients attending the Department of Dermatology, Princess Alexandra Hospital, Brisbane, Australia. It was undertaken with reference to the National Statement on Ethical Conduct in Human Research (2007; updated 2018). Human research ethics and research governance approval were sought, and approval was granted by the Metro South Human Ethics Committee (HREC/18/QPAH/245‐SSA/18/QPAH/246) and the University of Queensland Office of Research Ethics (#2018001616) and (#2022/HE000719).

### 2.5 ELISA Assay

Cytokine concentrations of IL‐17A, TNF, IL‐23, and IL‐22 were quantified using DuoSet ELISA kits (R&D Systems; DY317 for IL‐17A, DY210 for TNF, DY1290 for IL‐23, and DY782 for IL‐22) to validate measurements obtained from the digital SERS nanopillar assay. All assays were performed according to the manufacturer's protocols. ELISA‐derived concentrations were compared directly with those obtained using the digital SERS nanopillar platform.

### 2.6 SERS Nanopillar Assay

To minimize variability and ensure equal protein load per assay, skin biopsy lysates were normalized to protein mass of ∼13.84 µg (as determined by Pierce BCA Protein Assay) prior to analysis. This protein amount falls within the range obtainable from minimally invasive skin sampling methods, such as tape stripping (typically ∼1–10 µg protein per strip) and microbiopsy or suction blister techniques, which can yield tens of micrograms depending on sampling depth and anatomical site [45, 46]. The normalized sample was incubated on the SERS nanopillar chip for 1 h at room temperature to allow cytokine capture by immobilized antibodies. It was then sequentially washed with 0.5% Tween 20 wash buffer followed by two washes with PBS and finally Milli Q water to remove unbound components.

The Raman mapping was performed using the WITec Alpha 300R (ANFF, UQ node, QLD) with Laser wavelength = 633 nm, magnification = 100X, integration time = 0.01 s. The nine 60 × 48 µm Raman‑mapping regions were selected using a predefined, evenly spaced grid pattern across the nanopillar array to ensure representative spatial sampling and minimize sampling bias [40]. The detection threshold was determined by analyzing Raman signals obtained from negative control samples (i.e., sample devoid of cytokines) and active nanopillars were defined active nanopillars as those exhibiting reporter‐specific Raman intensities that were three times the background noise level within the defined spectral window. This thresholding approach enabled reliable discrimination between true reporter signals and background fluctuations during digital counting [40].

## 3 Results & Discussion

### 3.1 SERS Nanopillar Assay for Trace Cytokine Profiling of Skin Biopsy Samples

**FIGURE 1:**
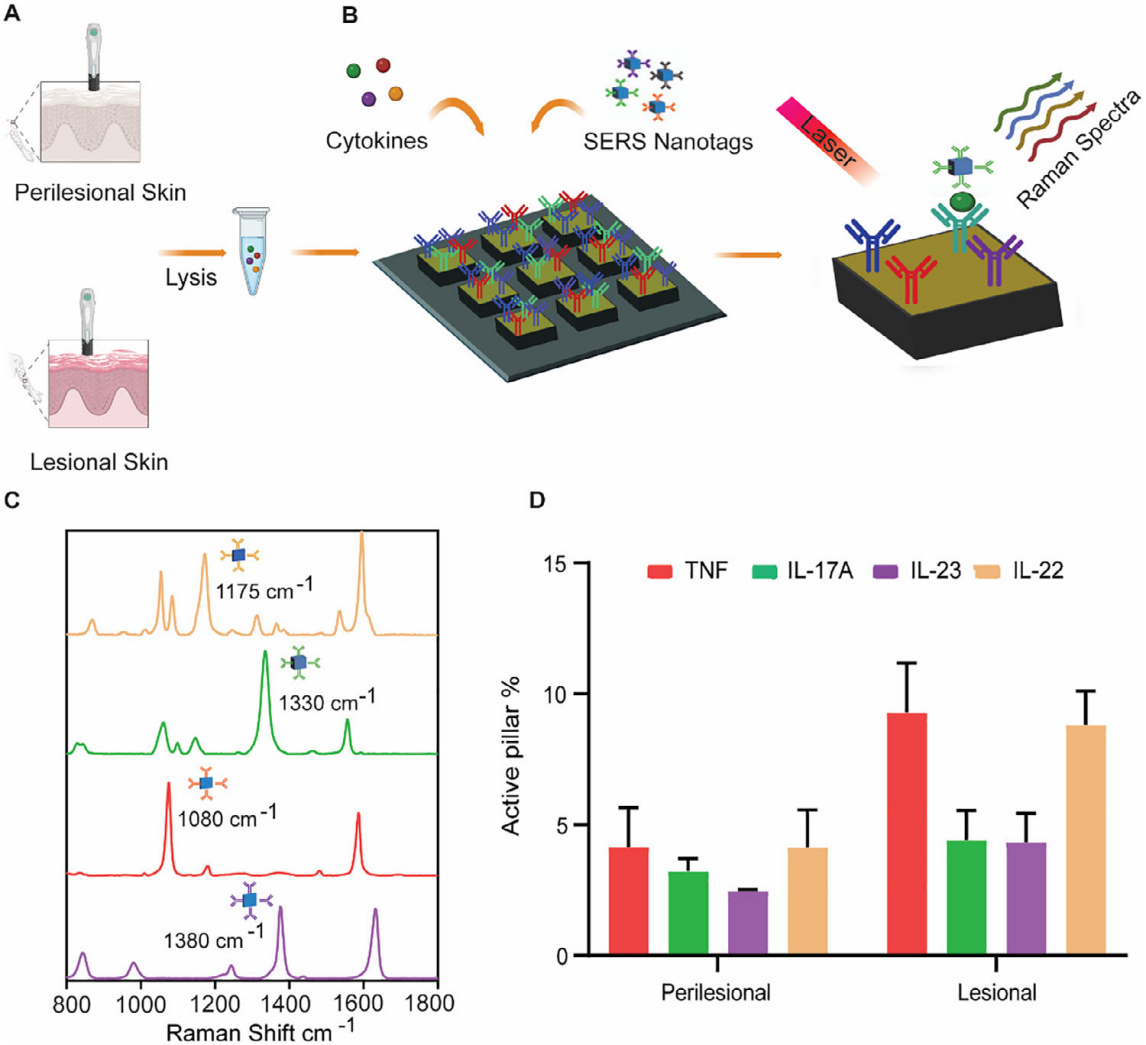
Digital SERS nanopillar assay for cytokine profiling in psoriasis. (A) Schematic illustration of skin samples collected from lesional and perilesional areas of a patient using a punch biopsy (B) Working of the digital SERS nanopillar assay (C) SERS mapping reveals characteristic Raman signals at 1175 cm^−1^ for MMC which conjugates to IL‐22, 1330 cm^−1^ for DTNB which conjugates to IL‐17A, 1080 cm^−1^ for MBA which conjugates to TNF and 1380 cm^−1^ for TFMBA which conjugates to IL‐23. (D) Example representation of average characteristic Raman signals to provide cytokine profiles in perilesional and lesional skin samples.

The analysis of pro‐inflammatory cytokines in skin samples of patients with psoriasis has clinical relevance to guided treatment [47]. In the context of inflammatory skin diseases, several studies have examined both lesional and perilesional skin samples from patients to investigate cytokine level variations. Perilesional skin, which appears morphologically normal at the time of biopsy, can still exhibit elevated levels of pro‐inflammatory cytokines [25, 48]. This may indicate the early stages of an immune response, potentially serving as a precursor prior to the physical manifestation of inflammation. If lesional skin already displays morphological changes associated with inflammation, cytokine profiling in these areas can provide insights into the severity and progression of the disease. These observations underscore the importance of spatial profiling of cytokines in subclinical inflammatory skin and in understanding the dynamic immune environment during disease progression [25]. While skin microsampling techniques may be suitable for frequent sampling and collection of multiple areas at the same time from skin lesional and perilesional sites, the limited quantities of extracted skin material require methods with high detection sensitivity for cytokine profiling,

In this study, we accessed archived lesional and perilesional psoriasis skin samples obtained from a 2 mm punch biopsy as previously described [44] (Figure 1A). To accurately profile trace level cytokines in skin biopsies, we designed a single‐molecule sensitive SERS immunoassay that used a nanopillar array and plasmonic nanotags for SERS read‐out (Figure 1B). This nanopillar array consisted of 250 000 pillars, and by following Poisson distribution, 10% of the pillars were activated (Active Pillar %) with the target cytokine on it, enabling single molecule detection. The antibody‐functionalized nanopillar array acted as a capture compartment for cytokines to form single‐molecule immune complexes, once labelled with the single particle active SERS nanotags [40]. To enable cytokine identification, we used the following Raman reporter/antibody pairing to prepare the SERS nanotags: MMC/anti‐IL‐22, DTNB/anti‐IL‐17A, TFMBA/anti‐IL‐23, TFMBA/ anti‐IL‐23, and MBA/anti‐TNF. These SERS nanotags provided characteristic Raman peaks at 1175, 1330, 1380, and 1080 cm^−1^, correspondingly (Figure 1C). We hypothesized that accurate trace cytokine profiling in skin biopsy samples could differentiate perilesional and lesional samples of patients with inflammatory skin disease (Figure 1D).

### 3.2 Specificity of SERS Nanopillar Assay

**FIGURE 2:**
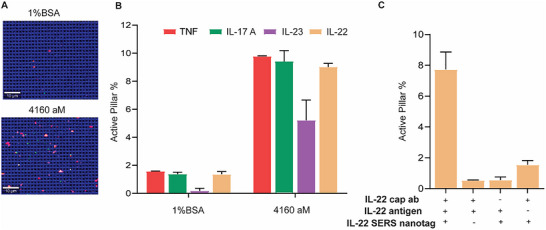
Specificity of digital SERS nanopillar assay for cytokine profiling. (A) False‐color Raman image of negative control and 10% (4160 am) cytokines spiked in 1% BSA in PBS. (B) Bar graph plots illustrate the specificity of the SERS nano assay in detecting four target cytokines: TNF, IL‐23, IL‐22, and IL‐17A. Each bar represents the mean active pillar percentage ± standard deviation for TNF (red), IL‐23 (violet), IL‐22 (brown), and IL‐17A (green) obtained from three technical replicates (*n* = 3) (*p*<0.005). (C) Active pillar percentage for IL‐22 under conditions shown in the figure: presence or absence of IL‐22 capture antibody, antigen, and SERS nanotag. Bar graph represents average Raman peak intensity with error bars showing the standard deviation obtained from three technical replicates.

The ability of the SERS nanopillar assay to generate specific cytokine profiles is critical for monitoring treatment responses in conditions such as psoriasis. To assess the assay's specificity for detecting TNF, IL‐22, IL‐17A, and IL‐23, we analyzed negative control samples devoid (i.e., 1% BSA in PBS) of cytokines and a positive control sample (i.e., a cocktail of the four cytokines at a concentration of 4160 aM), which is expected to yield approximately 10% active pillars. Figure 2A presents representative false‐color Raman images, where Au and Si appear as black and blue, respectively, and the SERS signals for TNF, IL‐22, IL‐17A, and IL‐23 are visualized in red, brown, green, and violet, respectively. The negative control exhibited minimal Raman signals across all cytokines, indicating low non‐specific binding. In contrast, the positive samples produced distinct and elevated Raman signals for each cytokine, consistent with specific targeted binding (Figure 2B). Positive samples yielded 5.5–9.8% active pillars, whereas the negative control consistently remained below 2%. A two‐tailed unpaired t‐test demonstrated a significant difference in signal between cytokine‐positive and negative controls (t (3) = 24.53, *p* = 0.0017), with the mean response for the target cytokine group (9.36) being markedly higher than the non‐target group (1.60). The difference between means was 7.76 ± 0.32 (95% CI: 6.40–9.12), corresponding to a very large effect size (R^2^ = 0.997), confirming high assay specificity across the four cytokines tested.

Next, we tested our assay for cross‐reactivity using IL‐22 as an example (Figure 2C). Here, a sample containing 4160 am of IL‐22 was prepared and detected using the corresponding IL‐22‐specific capture and detection antibodies. The cross‐reactivity of the system was further tested against negative controls that included: i) non‐target SERS nanotag (against IL‐6), and ii) non‐target capture antibody (against IL‐6), and non‐target cytokine (IL‐6). In each of these control scenarios, negligible background signals were observed, confirming the minimal nonspecific binding. Collectively, these experiments underscore the assay's high specificity and minimal background interference, which are two critical attributes for it'As successful application in clinical samples and multiplex biomarker profiling within complex tissue environments.

### 3.3 Sensitivity of the SERS Nanopillar Assay

**FIGURE 3:**
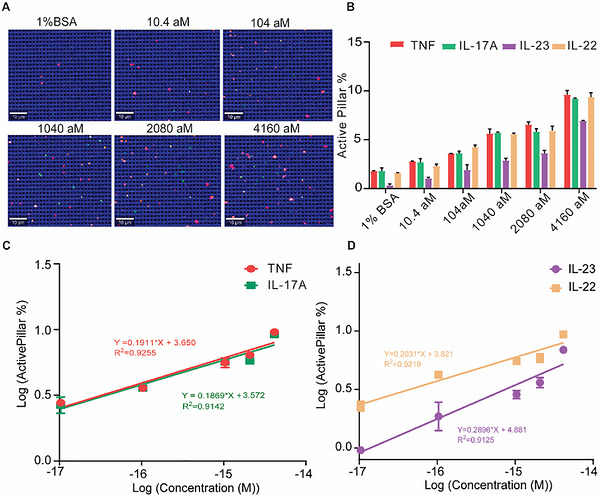
Sensitivity analysis of the digital SERS nanopillar assay for multiplexed cytokine detection. A) Representative false‐color Raman images obtained from samples containing TNF, IL‐23, IL‐22, and IL‐17A at concentrations of 10.4 to 4160 am. The corresponding bars represent the mean active pillar percentage ± standard deviation from three technical replicates (*n* = 3). C) Regression line that shows the R^2^ Values of the digital SERS nanopillar assay for TNF and IL‐17A cytokines at varying concentrations. D) Regression line that shows the R^2^ Values of the digital SERS nanopillar assay for IL‐23 and IL‐22 cytokines at varying concentrations.

Cytokines are typically present at very low concentrations (<2 pg mL^−1^) in clinical samples, making it essential to use highly sensitive platforms for their detection [49]. Accurate cytokine profiling is critical for evaluating patient immune responses and measuring the outcomes of treatment strategies. To investigate the limit of detection and dynamic range for the multiplexed cytokine detection of the SERS nanopillar assay, we titrated solutions containing TNF, IL‐22, IL‐17A, and IL‐23 at 10.4 aM to 4160 aM to comply with the Poisson distribution and recorded the active pillar percentage. Specifically, we added approximately 6.25 × 10^1^, 6.25 × 10^2^, 6.25 × 10^3^, 1.25 × 10^4^, 2.5 × 10^4^ cytokine molecules/10 µl on the nanopillar array, which should theoretically yield 0.025%, 0.25%, 2.5%, 5% and 10% of activated pillars, respectively. Figure 3A shows representative false‐color Raman images that indicate a proportional signal increase with increasing cytokine concentrations. Similarly, for quantitative measurement of cytokine profiles, the averaged Raman peak signal (Figure 3B) and calibration curves (Figure 3C,D) indicate a strong linear relationship between log_10_‐transformed concentration and log_10_‐transformed active pillars with a coefficient of determination (R^2^) over 0.90. The dynamic range covered two orders of magnitude and enabled trace cytokine detection with a limit of detection of ∼104 aM (based on three times the standard deviation of the blank) for the four target cytokines, which corresponds to approximately 1.5–1.9 pg mL^−1^. Cytokine concentrations reported in psoriatic skin biopsies typically range from tens to several hundred pg/mL for key inflammatory mediators such as TNF, IL‐17A, IL‐23, and IL‐22, indicating that the analytical sensitivity of the digital SERS nanopillar assay is well below the concentrations commonly observed in inflamed skin tissue [50, 51]. Even though these cytokines are present in abundance in the lesional skin, such sensitivity may be particularly advantageous when analyzing highly diluted lysate samples, small biopsy specimens, or minimally invasive microsampling approaches, where cytokine concentrations may be substantially lower [19, 45, 52]. It should be noted that the regression curves were separated due to the difference in signal strength obtained at different concentrations of cytokines for the different Raman reporters.

After establishing the dynamic range for cytokine detection, we were interested in assessing chip‐to‐chip reproducibility to account for potential quantification biases across different batches of SERS nanopillar assays. Cytokine detection was performed on four independent nanopillar chips using a sample containing all four cytokines at a concentration of 4160 aM. The platform showed good chip‐to‐chip reproducibility, with RSD values of 5.65% (TNF), 17.52% (IL‐17A), 15.64% (IL‐23), and 14.3% (IL‐22), confirming consistent assay performance across independent chips (Table S1).

**FIGURE 4:**
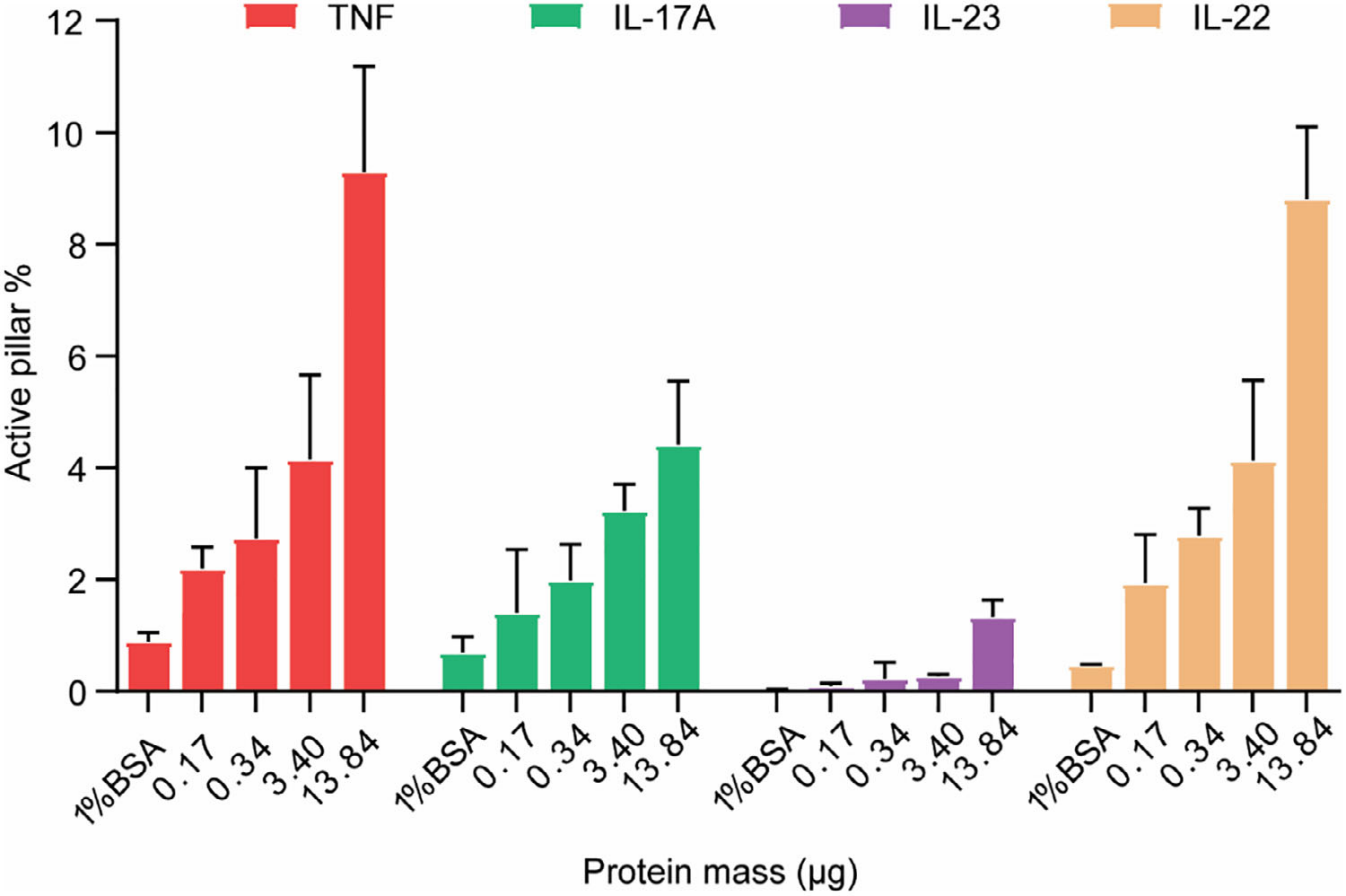
Sensitivity analysis of the digital SERS nanopillar assay using serially diluted clinical punch biopsy samples. Protein masses used in this study were 13.84, 3.4, 0.34, and 0.17 µg. The bar graphs represent average Raman peaks of three technical replicates and error bars show the standard deviation.

While clinical punch‑biopsy samples typically yield total protein masses in the tens of microgram range (∼13.84 µg), only a fraction of the total protein mass will be the cytokines, rendering their reliable quantification challenging [53]. We therefore selected this protein mass to evaluate whether the SERS nanopillar assay is applicable for cytokine detection under clinically relevant sample‑limited conditions. In comparison, minimally invasive microsampling techniques such as microbiopsy or tape stripping often provide even less material, with tape stripping commonly yielding <10 µg protein per strip [45]. To further assess whether the detection sensitivity of the digital SERS nanopillar assay is suitable for cytokine profiling in lesional and perilesional punch biopsy samples at very lower scales, we analyzed a serial dilution of a clinical punch biopsy sample from psoriasis patients (total protein mass = 13.84 µg) and recorded the active nanopillar percentage. Protein masses of 13.84, 3.46, 0.34, and 0.17 µg were tested. As shown in Figure 4, active pillar percentages increased with increasing protein mass. While cytokine levels varied across targets (e.g., TNF > IL‐23), the assay reliably detected cytokines at input levels as low as 0.34 µg. A noticeable decrease in cytokine levels was observed in the case of IL‐23. This reduction could be due to IL‐23 expression in this clinical skin sample being inherently low. These results demonstrate the high sensitivity and dynamic range of the digital SERS nanopillar assay, supporting its use for multiplex cytokine analysis in clinically relevant, low‐input samples such as tape stripping or other microsampling techniques [52, 54].

To validate the digital SERS nanopillar assay against a gold‐standard immunoassay, cytokine measurements were compared with an ELISA using a patient‐derived skin lysate samples, analyzed both in the absence and presence of recombinant cytokine spike‐in. In the non‐spiked lysate samples, the ELISA did not detect IL‐22, IL‐17A, IL‐23, or TNF, indicating that the analyte levels were below the assay limit of detection (Table S2). In contrast, the digital SERS nanopillar assay detected all four cytokines, with measured concentrations of 10.61 ± 4.84 pM for IL‐22, 11.89 ± 3.46 pM for IL‐17A, 2.34 ± 0.35 pM for IL‐23, and 11.52 ± 2.61 pM for TNF. These findings highlight the higher analytical sensitivity of the digital SERS nanopillar platform for cytokine detection in low‐input clinical samples.

In cytokine spike‐in lysates, both ELISA and the digital SERS nanopillar assay detected all analytes within a comparable concentration range and in the same overall order of magnitude (Table S2). The differences observed in absolute concentrations between the two methods likely arise from multiple factors, including differences in assay format, calibration strategy, antibody binding affinity, and susceptibility to matrix effects in complex skin lysate samples. In addition, ELISA provides an ensemble signal averaged across the sample, whereas the digital SERS nanopillar assay is based on Poisson‐guided digital event counting, which may further contribute to differences in absolute quantification. Overall, these results demonstrate that the digital SERS nanopillar assay enables cytokine quantification in a clinically relevant concentration range while offering improved sensitivity in non‐spiked lysates where ELISA measurements were below the limit of detection. Nevertheless, further validation in larger cohorts and through additional orthogonal methods will be necessary to establish the extent of quantitative agreement between the two platforms.

### 3.4 Cytokine Profiling in Naïve Psoriasis Punch Biopsy Samples

**FIGURE 5:**
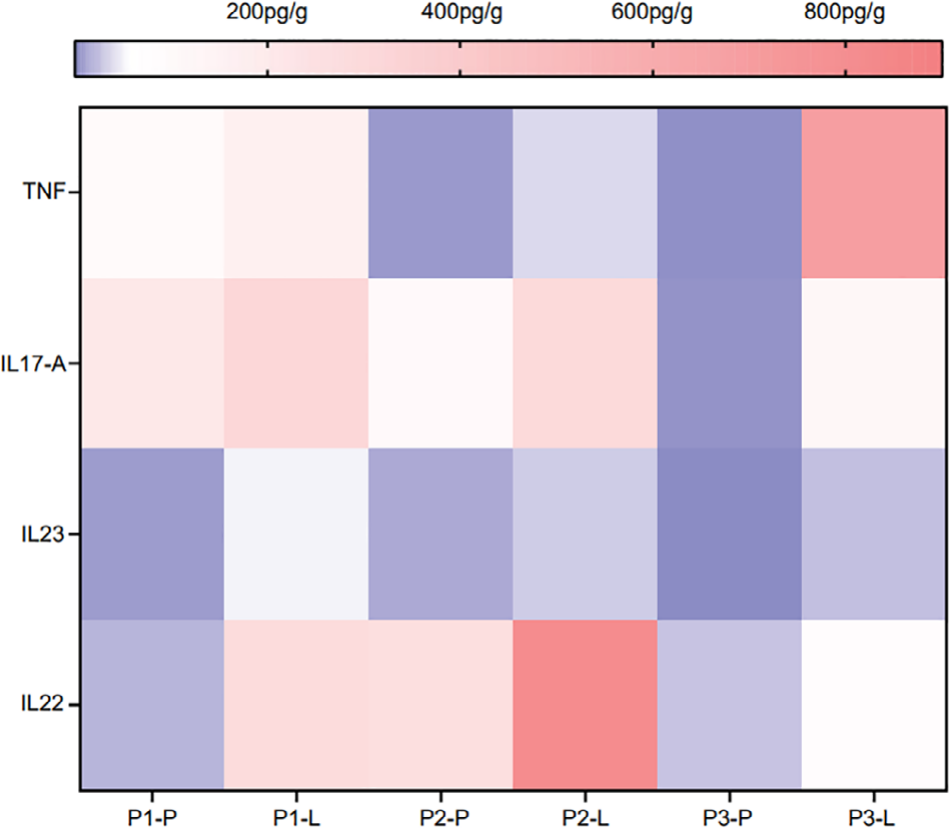
Heatmap representation of cytokine expression (pg/g) in lesional (L) and perilesional (P) skin biopsies from psoriasis patients (P1, P2, P3) (technical replicates *n* = 3; biological replicates *n* = 3), as quantified using the digital SERS nanopillar assay. Rows correspond to specific cytokines (TNF, IL‐17A, IL‐23, and IL‐22), while columns represent individual biopsy samples. Color intensity reflects relative cytokine concentration, as indicated by the color scale bar. The heatmap is presented for qualitative visualization only and is not intended for quantitative interpretation or inference of statistical significance or population‐level trends, given the limited sample size.

To quantify the presence of cytokines in the psoriatic skin biopsies from treatment‐naïve patients using a digital SERS nanopillar assay, we used three lesional and matched non‐lesional skin samples from the same patients (Table S3). The analysis revealed both inter‐ and intra‐patient variability in cytokine expression. As shown in Figure 5, IL‐17A exhibited the most pronounced elevation, with mean levels increasing from approximately 120 pg g^−1^ in perilesional samples to approximately 650 pg g^−1^ in lesional tissue. TNF concentrations rose from approximately 85 pg g^−1^ in perilesional to 410 pg g^−1^ in lesional, highlighting strong pro‐inflammatory signaling in psoriatic lesions. IL‐23 levels increased from approximately 10 to 50 pg g^−1^.

Paired statistical analysis revealed significantly elevated cytokine levels in lesional compared to perilesional skin across all patients (Table S4). These differences were supported by moderate‐to‐large to large effect sizes (TNF: *d* = 0.79; IL‐17A: *d* = 1.66; IL‐23: *d* = 1.57; IL‐22: *d* = 1.42), indicating a consistent increase in inflammatory mediators despite inter‐patient variability. These results are consistent with previously reported cytokine profiles associated with activation of the IL‐23/Th17 axis when comparing perilesional to lesional samples [55]. IL‐22 displayed a more moderate increase, from approximately 20 pg g^−1^ to approximately 400 pg g^−1^, reflecting its known role in epithelial functions and keratinocyte proliferation in lesional and perilesional samples. Overall, these multiplexed cytokine patterns suggest a heightened Th17‐associated inflammatory response in lesional compared to perilesional skin. However, given the limited cohort size (*n* = 3 paired biopsies), these observations should be interpreted as preliminary findings within a proof‐of‐concept study and that the heatmap provides a qualitative overview of cytokine patterns across patients; it is not intended for quantitative interpretation. Further studies with a large cohort will account for inter‐individual variability and more accurately characterization of disease‐associated cytokine landscapes.

To assess potential matrix interference from complex skin biopsy lysates, spike‐and‐recovery experiments were performed using a microbiopsy‐derived perilesional skin sample normalized to 0.34 µg total protein. Recombinant cytokines were spiked at a level corresponding to 1040 aM, yielding recovery values of 91.0% (TNF), 97.7% (IL‐17A), 121.2% (IL‐23), and 116.5% (IL‐22), indicating minimal matrix interference under the assay conditions (Table S5).

It should be noted that the digital SERS platform developed in this study is more sensitive than previously reported platforms that are being used for cytokine detection in skin diseases, while also providing multiplexing capability. As summarized in Table 1, the digital SERS nanopillar assay provides several practical advantages compared with existing cytokine detection technologies, providing similar assay turnaround times (∼2–4 h) [56, 57, 58]. The platform enables ultrasensitive detection (104 aM, as low as ∼ 1.8 fg mL^−1^) while simultaneously quantifying multiple psoriasis‐associated cytokines (IL‐22, IL‐17A, IL‐23, and TNF). In contrast, fluorescence bead assays, surface plasmon resonance, and electrochemiluminescence methods typically have limits of detection in the pg/mL to ng/mL range. Importantly, the assay is compatible with microscopically derived skin samples, making it particularly suitable for dermatological applications where tissue availability is limited. Future developments to to enable faster chip readouts could further support the translation of this assay into the clinical setting.

**TABLE 1:** Comparison of reported techniques for multiplex profiling of cytokines.

Detection	Principle	Targets	Turn Around Time	Limit of Detection	Ref
Digital SERS	Addition of single cytokines on nanopillar, labelled with SERS nanotags	IL‐22, IL17A, IL23, TNF	4 h	104am (∼ 1.8 fg mL^−1^)	This study
Fluorescence	Capture antibodies on distinct, fluorescence‐encoded beads bind specific target analytes	IL‐22, IL‐13^,^ Arginase, VEGF, CCL‐5	4 h	50 pg g^−1^	[44, 58]
Surface plasmon resonance	Analyte–antibody interaction changes refractive index at gold surface, shifting resonance signal proportional to bound analyte	IL‐17A	3.5 h	0.23 ng µL^−1^	[57, 59]
Electrochemiluminescence	Antibodies on carbon electrodes; SULFO‐TAG detection; electrical current induces light emission for quantification	IL‐1β, IL‐13, IL‐15, IL‐17A, IL‐18	2 h	1 pg mL^−1^	[56, 60]

Despite the promising analytical performance of the digital SERS nanopillar platform, several limitations should be acknowledged. From a technical perspective, further work will be required to evaluate the scalability of the assay to render the SERS nanopillar assay amenable for potential clinical translation. From a biological perspective, cytokine measurements derived from skin biopsies may also be influenced by intrinsic tissue heterogeneity within a 2 mm punch biopsy, where immune cell infiltration and cytokine expression can vary spatially across the lesion. Although the patients included in this proof‐of‐concept study were treatment‐naïve, cytokine profiles in broader clinical cohorts may be influenced by factors such as prior medication exposure, disease duration, and patient‐specific immune variability. Future studies involving larger patient cohorts and controlled clinical metadata will therefore be important to better understand the biological variability of cytokine profiles and further validate the clinical applicability of the digital SERS platform.

## 4 Conclusions

In this study, we developed a highly sensitive and specific digital SERS‐based nanopillar assay for the multiplexed detection of four key inflammatory cytokines (IL‐17A, IL‐22, IL‐23, and TNF) that are commonly overexpressed in psoriasis. This assay demonstrated excellent analytical performance, with a dynamic detection range spanning 104 aM (∼1.8 fg mL^−1^) to 4160 aM (∼70 fg mL^−1^). High specificity was achieved for all four cytokines with negligible cross‐reactivity, highlighting the assay's robustness for multiplexed profiling. This assay also revealed distinct cytokine expression profiles between lesional and perilesional sites, underscoring its potential utility in disease monitoring as well as providing insights into the mechanism of the disease at different lesion sites. However, given the limited cohort size in this proof‐of‐concept study (*n* = 3 patients), these observations should be interpreted as preliminary and not as definitive disease‐specific cytokine signatures. Larger studies will be required to fully evaluate patient heterogeneity, disease severity, and anatomical variation in cytokine expression

Furthermore, its application to microscopically derived skin samples containing as little as 0.34 µg of protein mass highlights the method's potential for minimally invasive diagnostics, paving the way for future clinical applications where tissue accessibility might be limited. Overall, the digital SERS nanopillar assay holds great promise which may enable precise and specific cytokine profiling with high sensitivity from biopsy samples, leading to personalized management of inflammatory skin diseases.

## Conflicts of Interest

The authors declare no conflicts of interest.

## Supporting information




**Supporting File**: smtd70693‐sup‐0001‐SuppMat.docx.

## Data Availability

The data that support the findings of this study are available from the corresponding author upon reasonable request.
