# Supplementary material for: Spatially Profiling Trace Cytokine Signatures From Microscopically Derived Skin Samples to Probe Skin Disease Inflammation
Source: Small Methods. 2026 May 12;10(13):e01964. doi: 10.1002/smtd.202501964 (PMC13353872; doi:10.1002/smtd.202501964)
Supplement: Supplementary file 1 — Supporting File: smtd70693‐sup‐0001‐SuppMat.docx. [file SMTD-10-e01964-s001.docx]

Supplementary Information

**Spatially profiling trace cytokine signatures from microscopically derived skin samples to probe skin disease inflammation**

Aditya Krishnakumar^1^, Zhen Zhang^1^, Courtney Vedelago^1^, Timothy J. Liu^2,3^, Yung-Ching Kao^2^, H. Peter Soyer^2,3^, Mitchell S. Stark^2^, Snehlata Kumari^2*#^, Matt Trau^1,4*#^Alain Wuethrich^1,4*#^

^1^Centre for Personalised Nanomedicine Australian Institute for Bioengineering and Nanotechnology (AIBN). The University of Queensland, Brisbane, QLD 4072, Australia

^2^Frazer Institute, Faculty of Health, Medicine and Behavioural Science, The University of Queensland, Brisbane, Queensland 4102, Australia

^3^Department of Dermatology, Princess Alexandra Hospital, Brisbane, QLD, Australia

^4^School of Chemistry and Molecular Biosciences, The University of Queensland, Brisbane, QLD 4072, Australia.

*Authors contributed equally.

#co-corresponding authors.

Email addresses: s.kumari@uq.edu.au, m.trau@uq.edu.au and a.wuethrich@uq.edu.au

**Supplementary Table 1**. Detection of cytokines on four independent chips with 4.16 fM of cytokines.

| Cytokines | Chip 1 (fM) | Chip 2 (fM) | Chip 3 (fM) | Chip 4 (fM) | RSD (%) |
| --- | --- | --- | --- | --- | --- |
| TNF | 5.06 | 4.47 | 4.72 | 4.53 | 5.65 |
| IL-17A | 3.08 | 4.3 | 4.35 | 4.75 | 17.52 |
| IL-23 | 3.8 | 3.3 | 3.82 | 2.69 | 15.64 |
| IL-22 | 3.5 | 4.86 | 4.81 | 4.5 | 14.3 |

**Supplementary Table 2.** Comparison of SERS nanopillar assay and ELISA in unspiked and spiked patient sample (technical replicates, n=3). Not detectable, nd.

| Cytokine | ELISA (pM) | SERS (pM) | ELISA (pM) Spike in | SERS (pM) Spike in |
| --- | --- | --- | --- | --- |
| IL-22 | nd | 10.61±4.84 | 13.2±2.95 | 23.8±3.32 |
| IL-17A | nd | 11.89±3.46 | 6.3±0.15 | 22.1±4.64 |
| IL-23 | nd | 2.34±0.35 | 59.8±5.70 | 15.1±1.60 |
| TNF | nd | 11.52±2.61 | 8.4±0.12 | 20.2±2.19 |

**Supplementary Table 3.** Patient skin samples.

| Patient ID | Sample Type | Treatment | Site of Biopsy | Lesional appearance |
| --- | --- | --- | --- | --- |
| P1-P | Punch Biopsy | No treatment | R) forearm | Normal |
| P1-L | Punch Biopsy | No treatment | R) forearm | Affected |
| P2-P | Punch Biopsy | No treatment | L) forearm | Affected |
| P2-L | Punch Biopsy | No treatment | L) forearm | Affected |
| P3-P | Punch Biopsy | No treatment | R) middle back | Normal |
| P3-L | Punch Biopsy | No treatment | R) middle back | Affected |

**Supplementary Table 4**. Statistical analysis of lesional vs. perilesional samples. p‑values were calculated using a paired, two‑tailed t‑test comparing cytokine levels between lesional and perilesional skin samples for each patient (technical replicates, n = 3). Confidence intervals represent the 95% confidence interval of the mean difference. Significance threshold: p < 0.05.

|  | Patient 1  (p values) | CI (pg/g) | Patient 2  (p values) | CI (pg/g) | Patient3  (p values) | CI (pg/g) | Cohen’s d  (Paired) | Effect Size |
| --- | --- | --- | --- | --- | --- | --- | --- | --- |
| IL17A | 0.0006* | 256.8 to 319.3 | 0.0005* | 188.4 to 230.5 | 0.0091* | 51.70 to 124.6 | 1.66 | Large |
| TNF-α | 0.015* | 26.28 to 86.43 | 0.015* | 9.956 to 52.06 | 0.015* | 52.28 to 730.3 | 0.79 | Moderate-Large |
| IL23 | 0.015* | 110.3 to 129.6 | 0.0009* | 31.91 to 41.53 | 0.0077* | 35.93 to 79.81 | 1.57 | Large |
| IL22 | 0.0004* | 247.8 to 292.9 | 0.0055* | 340.3 to 661.7 | 0.0037* | 54.21 to 92.83 | 1.42 | Large |

**Supplementary Table 5**. Assessment of matrix interferences. Recovery test of four cytokines spiked in human skin lysate (0.34 μg protein mass) and analysed by the SERS nanopillar assay.

| Cytokines | Added (fM) | Detected (fM) ±SD | Recovery (%) | RSD (%) |
| --- | --- | --- | --- | --- |
| TNF | 1.040 | 2.02±0.21 | 90.98 | 10.82 |
| IL-17A | 1.040 | 2.21±0.46 | 97.72 | 21.00 |
| IL-23 | 1.040 | 1.51±0.16 | 121.15 | 10.59 |
| IL-22 | 1.040 | 2.38±0.33 | 116.50 | 13.98 |
